# Cornea‐SELEX for aptamers targeting the surface of eyes and liposomal drug delivery

**DOI:** 10.1002/EXP.20230008

**Published:** 2024-02-09

**Authors:** Ka‐Ying Wong, Yibo Liu, Man‐Sau Wong, Juewen Liu

**Affiliations:** ^1^ Department of Chemistry, Waterloo Institute for Nanotechnology University of Waterloo Waterloo Ontario Canada; ^2^ Centre for Eye and Vision Research (CEVR) 17 W Hong Kong Science Park Hong Kong Hong Kong; ^3^ Department of Food Science and Nutrition The Hong Kong Polytechnic University Hung Hom, Kowloon Hong Kong; ^4^ Research Center for Chinese Medicine Innovation The Hong Kong Polytechnic University Hung Hom, Kowloon Hong Kong SAR P. R. China

**Keywords:** aptamers, cornea, tissue‐SELEX

## Abstract

Cornea is the major barrier to drug delivery to the eye, which results in low bioavailability and poor efficacy of topical eye treatment. In this work, we first select cornea‐binding aptamers using tissue‐SELEX on pig cornea. The top two abundant aptamers, Cornea‐S1 and Cornea‐S2, could bind to pig cornea, and their *K*_d_ values to human corneal epithelial cells (HCECs) were 361 and 174 nм, respectively. Aptamer‐functionalized liposomes loaded with cyclosporine A (CsA) were developed as a treatment for dry eye diseases. The *K*_d_ of Cornea‐S1‐ or Cornea‐S2‐functionalized liposomes reduces to 1.2 and 15.1 nм, respectively, due to polyvalent binding. In HCECs, Cornea‐S1 or Cornea‐S2 enhanced liposome uptake within 15 min and extended retention to 24 h. Aptamer CsA liposomes achieved similar anti‐inflammatory and tight junction modulation effects with ten times less CsA than a free drug. In a rabbit dry eye disease model, Cornea‐S1 CsA liposomes demonstrated equivalence in sustaining corneal integrity and tear break‐up time when compared to commercial CsA eye drops while utilizing a lower dosage of CsA. The aptamers obtained from cornea‐SELEX can serve as a general ligand for ocular drug delivery, suggesting a promising avenue for the treatment of various eye diseases and even other diseases.

## Graphical abstract

Using pig cornea as a target, aptamers were selected using the tissue‐SELEX method. By attaching the aptamers to liposomes loaded with cyclosporin A, improved treatment of dry eye disease was achieved in vitro and in vivo.

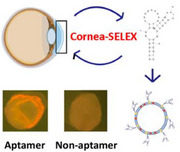

## 1 INTRODUCTION

Topical eye drops remain the most frequently used and easiest medication route for ocular drug administration and managing anterior eye diseases.^[^ ^1^ ^]^ However, various anatomic and physiologic barriers discourage the retention and active absorption of the drug, resulting in low bioavailability and poor efficacy.^[^ ^1^ ^]^ Thus, high concentration and repeated administration are required to reach desired therapeutic effects, which might lead to side effects and poor patient compliance.^[^ ^2^ ^]^ One of the main strategies to improve the ocular bioavailability of topical eye drops is to increase the retention time of the drug on the eye surface, which is the cornea.^[^ ^3^ ^]^ New techniques for delivering eye drops have emerged, such as prodrugs, cyclodextrins, in situ gels, and nanoparticles. These advancements are aimed at enhancing the absorption and distribution of drugs in both the front and back of the eye, thereby improving their bioavailability.^[^ ^4^ ^]^ However, active targeting nanomedicine in ocular therapeutics, which could reduce toxicity and adverse side effects, has been less addressed.^[^ ^5^ ^]^

Aptamers, which are single‐stranded oligonucleotides that can selectively bind to target molecules, could offer several advantages, including faster tissue penetration, low immunogenicity, tolerance to heat stress, ease of modification with different function groups and low‐cost synthesis.^[^ ^6^ ^]^ Additionally, aptamers have a wide range of biological targets, making them an attractive option for medicine, diagnostics, drug delivery and environmental monitoring.^[^ ^7^ ^]^ Aptamers have been studied as targeting ligand to promote therapeutic effects, but mainly in cancer tissues.^[^ ^6^, ^8^ ^]^

Interestingly, the first approved aptamer medication is an anti‐vascular endothelial growth factor (VEGF) aptamer for inhibiting the growth of abnormal blood vessels in age‐related macular degeneration and diabetic macular edema.^[^ ^9^ ^]^ The second aptamer‐based drug, which was approved just this year, is also for treating an eye disease called geographical atrophy. A PEGylated aptamer inhibiting platelet‐derived growth factor in age‐related macular degeneration patients has entered phase 3 clinical trial.^[^ ^10^ ^]^ Even though research on aptamer application in the eye is emerging, most of the studies focused on the posterior chamber of the eye,^[^ ^11^ ^]^ and few studies have been performed on ocular drug delivery targeting the eye surface, a major barrier to ocular drug delivery.

Liposomes have been widely used in drug delivery.^[^ ^12^ ^]^ They are vesicles composed of lipid bilayers, similar to the natural phospholipid membranes found in cells. Their unique structure allows them to encapsulate both hydrophilic and hydrophobic drugs, making them suitable carriers for a diverse array of therapeutic agents. They enhance drug solubility, protect drugs from degradation, and enable targeted delivery.^[^ ^12^ ^]^ Liposomes can provide sustained release, reduce side effects, and overcome biological barriers. Their versatility allows for customization in size, surface charge, and composition.^[^ ^12^, ^13^ ^]^ Earlier, we employed a previously reported mucin‐1 binding aptamer named S2.2 to deliver liposomes loaded with cyclosporine A (CsA), an FDA‐approved drug for dry eye diseases (DED), to human corneal epithelial cells.^[^ ^14^ ^]^ This study showed increased retention time and efficacy of the aptamer‐functionalized liposomes in cells under dry eye conditions, indicating the feasibility of aptamer liposomes as a delivery tool to the eye surface. However, the mucin aptamer was selected against mucin 1 peptides,^[^ ^15^ ^]^ which is not specific for eye tissues and may not bind optimally to the eye surface. Therefore, it is necessary to perform systematic evolution of ligands by exponential enrichment (SELEX) to select aptamers that directly target the eye surface.

The outermost surface of the eyes is the cornea, which is composed of corneal epithelial cells. While cell‐SELEX is very popular, especially in cancer research,^[^ ^16^ ^]^ it is a complex process and susceptible to artifacts due to dead cells.^[^ ^17^ ^]^ Aptamers selected on cells might not reflect the actual occurrence in vivo, hindering the aptamer application in physiological tissues.^[^ ^9^, ^18^ ^]^ Thus, tissue‐SELEX, which is performed on ex vivo tissue‐derived structures, might be a useful alternative in this case.^[^ ^19^ ^]^ Furthermore, in situ tissue slide‐based SELEX was applied to cancer tissues, like papillary thyroid carcinoma and breast cancer.^[^ ^20^ ^]^ To date, no aptamers have been selected for the surface of the eye or cornea using the tissue‐SELEX approach. We aimed to perform tissue‐SELEX to select DNA aptamers targeting the surface of the cornea, which could be applied in drug delivery to the cornea. Herein, we discovered two aptamers that could bind to both porcine eye and human corneal epithelial cells and possessed higher binding affinity to the cells than the previously reported mucin aptamer. We also demonstrated that the selected cornea aptamers could promote the retention time and therapeutic effect of CsA liposomes under dry eye conditions. An in vivo study was conducted to compare the effectiveness of cornea‐targeting CsA liposomes on DED management with Restasis®, a cyclosporine ophthalmic emulsion for patients with DED.^[^ ^21^ ^]^

## 2 RESULTS AND DISCUSSION

### 2.1 Cornea SELEX

**FIGURE 1:**
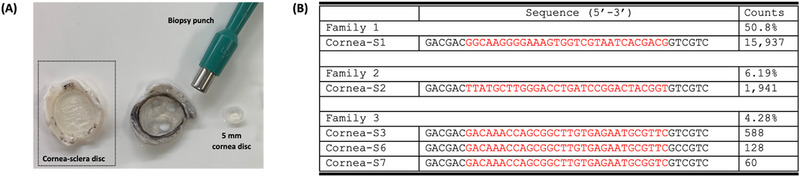
Tissue SELEX on pig cornea. (A) Cornea‐sclera tissues were isolated from pig eyes to maintain the integrity of the corneas. Corneal discs of 5 mm in diameter were then cut out by a biopsy punch. (B) The top three families from the cornea‐SELEX in the sequencing results of the round 12 enriched pool. The number of counts indicate the number of reads in the sequencing results. The N30 region is shown in red.

Aptamers have been investigated to facilitate targeted drug delivery in various tissues and cells.^[^ ^22^ ^]^ However, no aptamer was reported to target the cornea. Cornea has a multilayered structure, with the outermost layer being corneal epithelial cells. We isolated the corneas from pig eyeballs, and they have a hydrogel‐like appearance (Figure 1A). A DNA library with a 30‐nucleotide random region (N30), flanked by two constant primer‐binding regions, was used for aptamer selection. After incubation of the library (∼10^14^ random sequences) with a 5 mm corneal disc and extensive washing, 3 mm ethylenediamine tetra‐acetic acid (EDTA) was added to elute the bound DNA. EDTA‐chelated divalent metal ions, which are important to bind negatively charged DNA to cell surfaces.^[^ ^23^ ^]^ The eluted‐bound DNA strands were then amplified by PCR and collected for the next round of selection (Scheme S1).

A total of 12 rounds of selection were performed, and the last round of the enriched pool was sequenced. Out of the 31,344 sequences obtained, 155 unique sequences with more than two copies were obtained. The eight most abundant sequences could be divided into a few families (Figure 1B and Figure S1). Both families 1 and 2 contained only one sequence, which accounted for 50.8% and 6.19% of the total sequences, respectively. Family 3 contained three highly conserved sequences, which accounted for 4.48% of the total sequences. The number of counts in the other families was less than 1% of the total sequences. Given the complexity of the corneal surface, having a broad distribution of aptamer sequences was expected. In contrast, for small molecule targets, the number of distinct aptamers is often much less.^[^ ^24^ ^]^

### 2.2 Strong cell binding by aptamers

**FIGURE 2:**
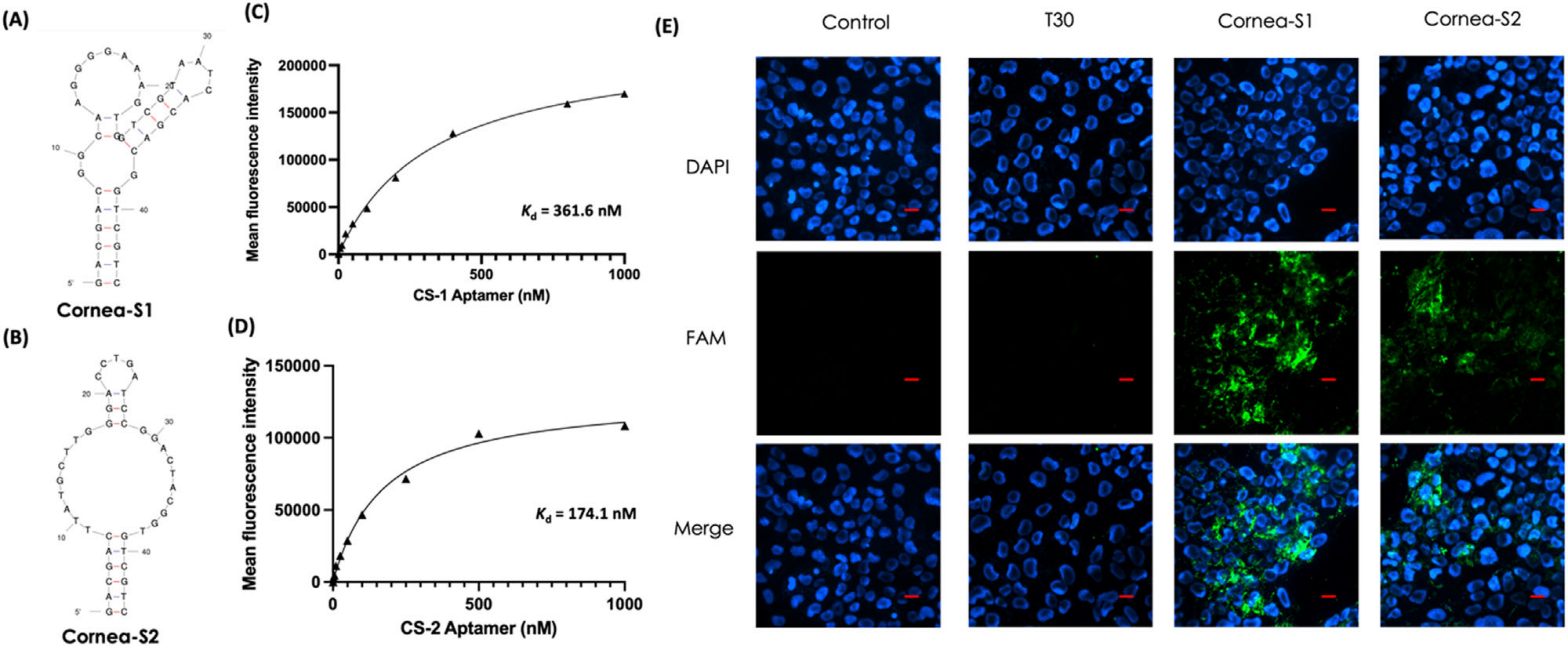
Binding of FAM‐labelled Cornea‐S1 and Corean‐S2 to HCECs. The secondary structures of (A) Cornea‐S1 and (B) Cornea‐S2 aptamers were predicted by Mfold. The *K*_d_ values of (C) Cornea‐S1 and (D) Cornea‐S2 in HCECs were calculated based on flow cytometry. *K*_d_ is the dissociatoin constant. (E) Fluorescence micrographs showing the binding ability of FAM‐labelled cornea aptamers in HCECs. The cell nucleus was stained with DAPI to give blue fluorescence, and the green fluorescence was from FAM‐labelled aptamers. Scale bars: 20 μm.

We decided to study the top two most abundant sequences, named Cornea‐S1 and Cornea‐S2 because they may have a higher binding affinity or may bind to the most abundant surface molecules on cornea.^[^ ^25^ ^]^ The secondary structures of Cornea‐S1 and Cornea‐S2 were predicted by Mfold, as shown in Figure 2A,B. Due to the large size of corneal tissue, it is difficult to do quantitative binding assays. Thus, we tested the binding of FAM‐labelled aptamer to human corneal epithelial cells (HCECs) using flow cytometry and fluorescence microscopy. BSA and salmon DNA were added to the blocking buffer to reduce non‐specific binding^[^ ^26^ ^]^ before the incubation with FAM‐labelled aptamers. In Figure 2C,D and Figure S2, both aptamers showed strong binding to HCECs. Cornea‐S1 and Cornea‐S2 had dissociation constant (*K*_d_) values of 362 and 174 nm, respectively. Also, both cornea aptamers had lower *K*_d_ values than the *K*_d_ value of the S2.2 mucin aptamer (515 nm) in HCECs (Figure S2), indicating the aptamers selected on cornea tissues had better recognition of eye cells compared to the mucin aptamer. These *K*_d_ values are not very low for aptamers reported to bind to cells. A potential reason could be that we used pig corneas for the selection, but the cell assays were based on human cells. Nevertheless, the binding affinity can be drastically improved by using polyvalent binding interactions (vide infra).

To understand the type of target, we treated HCECs with trypsin, which degraded surface proteins.^[^ ^27^ ^]^ Comparing the flow cytometry results between intact cells and trypsin‐treated cells, the latter showed significantly lower FAM signals, indicating Cornea‐S1 and Cornea‐S2 might target cell surface protein of the cornea (Figure S3). Moreover, we visualized the binding of FAM‐labelled aptamers in HCECs under a fluorescence microscope. In Figure 2E, no FAM signals were observed in the control and T30 DNA groups, but intensive FAM signals were observed in the Conrea‐S1 and Cornea‐S2 groups. In particular, the FAM signals in the Corena‐S1 group were observably higher than those in the Conrea‐S2 and S2.2 aptamers (Figure S2), implying that the expression of Cornea‐S1 targets might be higher than that of Cornea‐S2 targets in HCECs.

### 2.3 Strong polyvalent binding using aptamer‐functionalized liposomes

Next, aptamer‐functionalized liposomes were synthesized. The liposomes were made of DOPC (1,2‐dioleoyl‐sn‐glycero‐3‐phosphocholine) and Rhod‐PE (1,2‐dioleoyl‐*sn*‐glycero‐3‐phosphoethanolamine‐*N*‐(lissamine rhodamine B sulfonyl)) at a weight ratio of 99:1 with the CsA drug using the thin‐film hydration method as mentioned before.^[^ ^12^, ^14^ ^]^ The modification of liposomes with aptamers can be achieved either during their formation or by conjugating them to synthesized liposomes. When aptamers are conjugated during liposome formation, they are distributed on both the external and internal surfaces, potentially limiting the space for drugs.^[^ ^28^ ^]^ In our current study, we employed post‐insertion techniques, introducing cholesterol‐tagged aptamers into the lipid bilayer of synthesized liposomes. This approach offers customization benefits for preformed liposomes, allowing better control over aptamer distribution and drug‐loading capacity.^[^ ^28^, ^29^ ^]^

**FIGURE 3:**
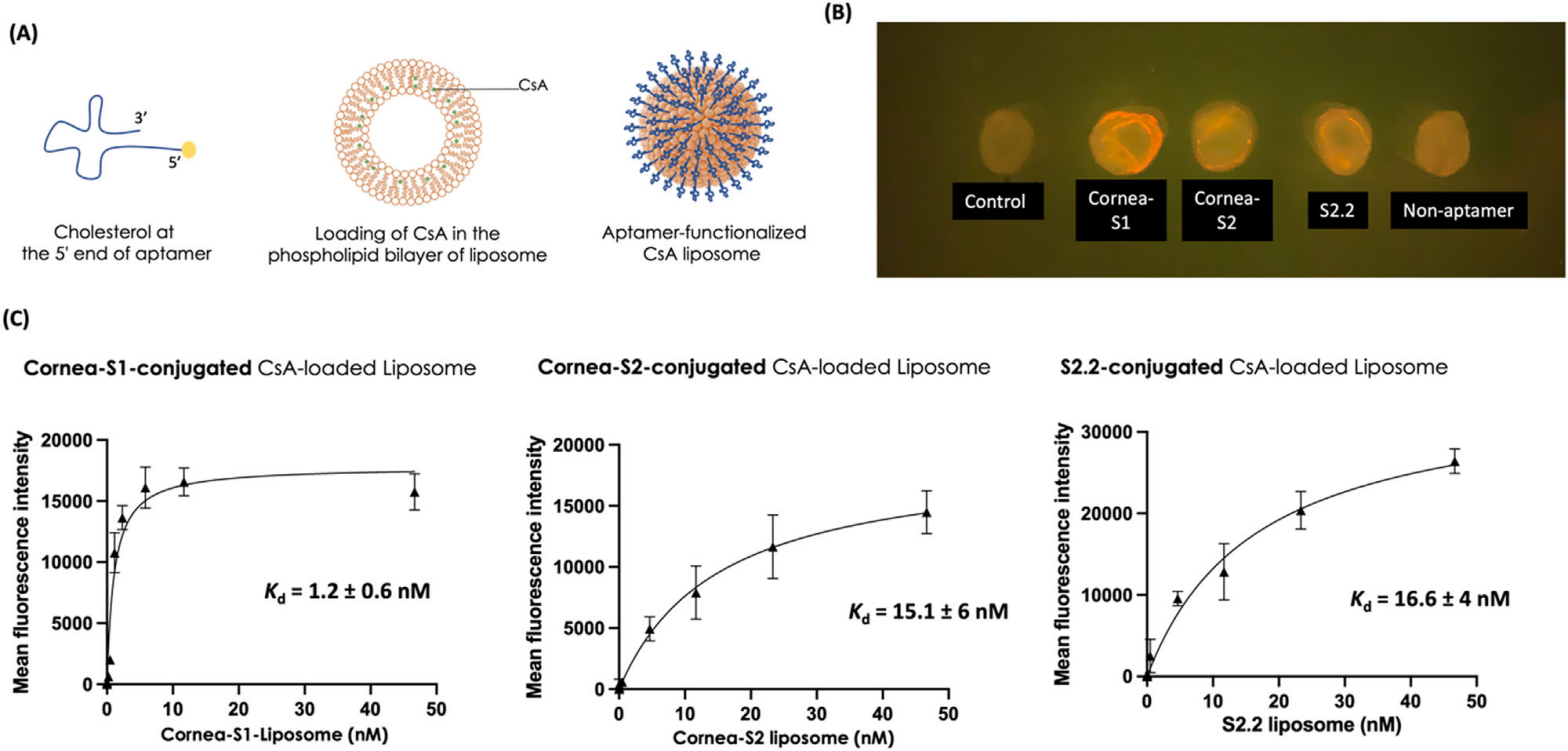
Binding of aptamer‐conjugated and CsA‐loaded liposomes to pig cornea and HCECs. (A) Cartoons showing a cholesterol‐conjugated aptamer, loading of CsA in the liposome, and an aptamer‐functionalized CsA‐loaded liposome. (B) A photograph showing the binding of aptamer‐functionalized rhodamine‐labeled CsA liposomes to pig cornea. PBS was used as the control. Scale bar: 5 mm. (C) Using flow cytometry, the evaluation of *K*_d_ values of aptamer‐conjugated and CsA‐loaded liposomes in HCECs. *K*_d_ is the dissociation constant. Control means liposomes without any DNA.

Based on dynamic light scattering, the liposomes have a mean particle size of 106 nm and a nearly neutral zeta potential.^[^ ^14^ ^]^ To investigate the effect of the selected cornea aptamers on the retention time and efficacy of CsA liposomes, we employed a non‐aptamer as the negative control sequence and an S2.2 aptamer as the positive control sequence. Non‐aptamer, S2.2, Cornea‐S1, or Cornea‐S2 aptamers were inserted into liposomes through the cholesterol at the 5′ end of the DNA strands (Figure 3A).^[^ ^30^ ^]^ After ultracentrifugation, the concentrations of free aptamer in the supernatant were measured by spectrophotometer, and each liposome had approximately 690 DNA strands (Figure S4). Moreover, we also assessed the size of liposomes after three months of storage at 4°C. The liposome size remained unchanged (106 nm), indicating the stable structure of liposomes in the refrigerator.

To evaluate the binding of aptamer‐functionalized liposomes, we incubated the Corena‐S1, Corena‐S2, or S2.2 Rhod‐labeled liposomes with freshly isolated pig cornea discs, followed by an extensive wash. In Figure 3B, there was no red fluorescent in the control group (liposomes without DNA attached), indicating low intrinsic red fluorescence of the cornea tissue and low nonspecific adsorption of liposomes to the cornea. The Corena‐S1 liposomes were found on the surface and mostly at the edge of the pig cornea, implying liposomal binding to the epithelial layer and the cross‐sectional area. The S2.2 or Corena‐S2 liposomes were found on the cornea surface but with weaker fluorescence. The fluorescence shown in these three groups was apparently greater than that in the non‐aptamer group, indicating the aptamers could recognize pig cornea. In addition, since these samples were imaged after extensive washing, these aptamer‐functionalized liposomes can achieve a long retention time on the cornea surface.

We then studied the binding affinity of each aptamer liposome in HCECs using flow cytometry. We incubated the cells with liposomes at room temperature instead of in a 37°C incubator to reduce the internalization of liposomes by endocytosis.^[^ ^31^ ^]^ In Figure 3C, Cornea‐S1‐liposomes had the lowest *K*_d_ values of 1.2 ± 0.6 nm compared to the Cornea‐S2 (15 ± 6 nm) or S2.2 (17 ± 4 nm) CsA liposomes. This apparent *K*_d_ is much lower than that observed in the free aptamers; for example, Cornea‐S1 being 300‐fold lower, and this was attributed to the stronger polyvalent binding increasing the avidity of the aptamer‐functionalized liposomes.^[^ ^32^ ^]^

### 2.4 Therapeutic efficacy of aptamer‐functionalized liposomes

**FIGURE 4:**
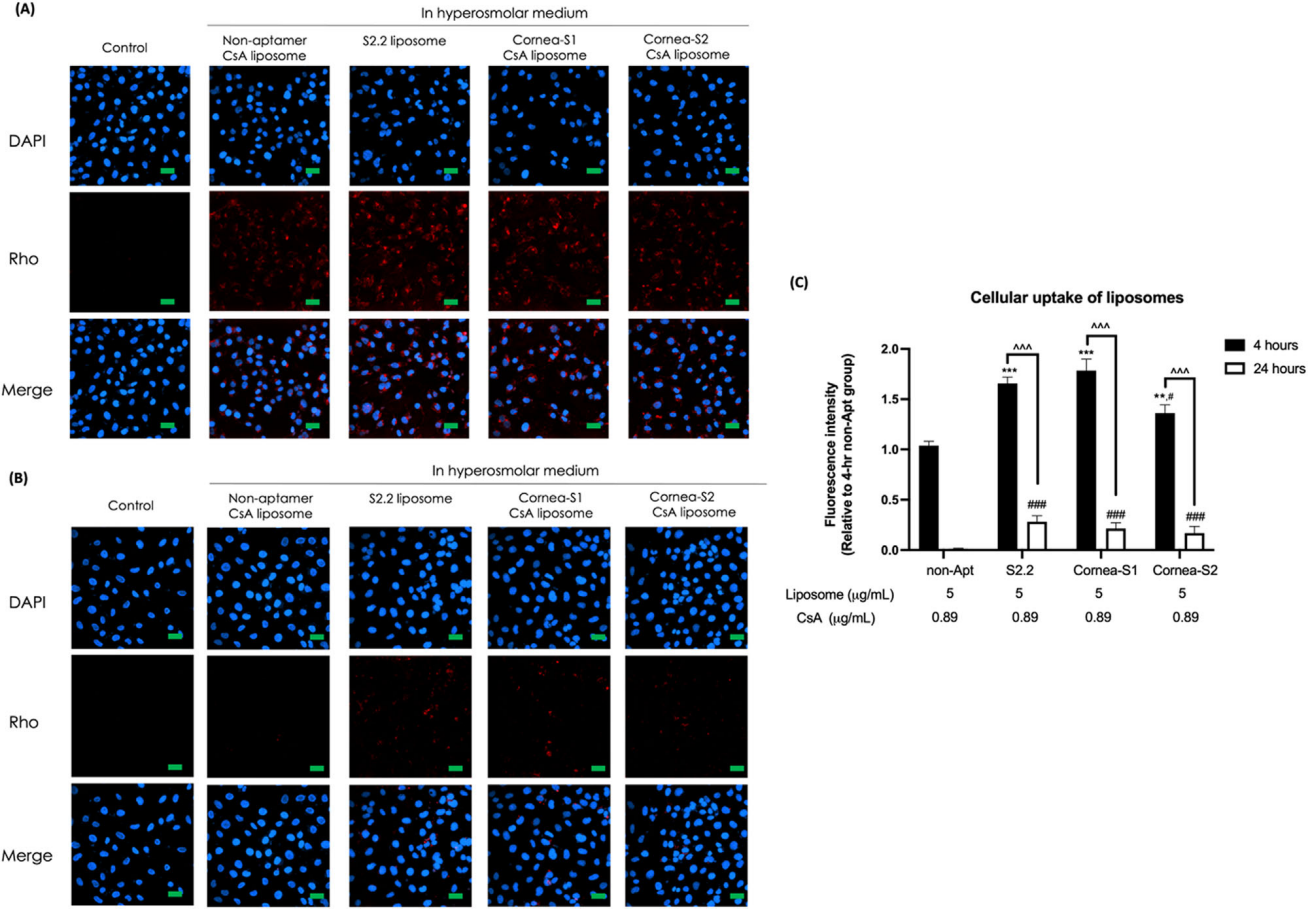
The retention of liposomes in dry eye HCECs model. (A) Fluorescence images of HCECs upon 4‐h incubation with aptamer liposomes. (B) Fluorescence images of HCECs upon 4‐h incubation with aptamer liposomes followed by 20‐h incubation in hyperosmolarity medium. The cell nucleus was stained with DAPI to give blue fluorescence and the red fluorescence (Rho) was from Rhod PE. Scale bar: 60 μm. (C) Quantification of fluorescence intensity. Data were shown as mean ± SEM and analyzed using One‐Way ANOVA analysis. *p < 0.05, **p < 0.01, ***p < 0.001 compared with DED group. Non‐apt: non‐aptamer control.

The development of DED is a complex process involving various factors, such as inflammation, tear film instability, and hyperosmolarity. Hyperosmolarity, which is characterized by an excess of solutes in tears, plays a significant role in the pathogenesis of the disease.^[^ ^33^ ^]^ We tested CsA‐loaded liposomes for cellular uptake and retention in a dry eye HCECs model induced by a high osmolarity medium. In Figures 4A,C, fluorescent imaging confirmed the cellular uptake of aptamer liposomes in 4 h at 37°C in HCECs under dry eye conditions. Such results were expected because incubating the cells at 37°C could allow endocytosis of DNA‐functionalized liposome by class A scavenger receptors or in a lipid‐raft–dependent, caveolae‐mediated manner.^[^ ^34^ ^]^ Most importantly, the fluorescent signals in cells treated with Cornea‐S1, Corena‐S2, or S2.2 CsA liposomes were observably and statistically higher than those in the non‐aptamer group, indicating aptamers enhanced the cellular uptake of liposomes. Also, the Cornea‐S1 liposomes had the highest cellular uptake in HCECs, potentially due to the better *K*_d_ values to HCECs. Furthermore, we tested the retention of liposomes in cells by pretreating the cells with liposomes for 4 h, which was followed by 20‐h incubation in the liposome‐free medium. The results in Figure 4B,C showed the presence of Cornea‐S1, Corena‐S2, or S2.2 aptamer CsA liposomes in HCECs after long incubation time, implying aptamer assessed the long retention of CsA liposomes.

It was reported that surface modification of liposomes could accelerate cellular uptake.^[^ ^35^ ^]^ We further investigated the kinetics of uptake. The cells were treated with non‐aptamer, Corean‐S1, or Cornea‐S2 CsA liposomes for 15 min, 30 min, 1 h, 2 h, and 4 h. The results indicated the presence of Conrea‐S1 and Cornea‐S2 aptamers allowed cellular uptake of liposome in 15 min and accumulation of liposome in 4 h in HCECs, while the non‐aptamer group required a longer incubation time (30 min) to have observable liposome uptake (Figure S5).

**FIGURE 5:**
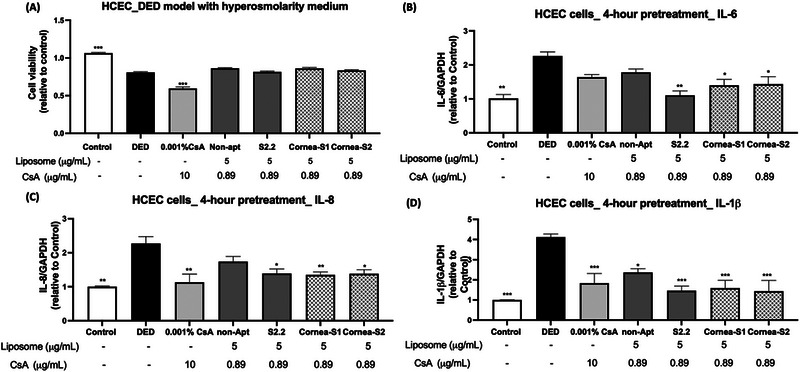
The efficacy of liposomes in the dry eye HCECs model. (A) The cell viability of HCECs upon CsA and liposome treatments for 24 h. (B–E) The gene expression of IL‐6, IL‐8 and IL‐1β. Data were normalized with GAPDH and relative to the control. Data were shown as mean ± SEM and analyzed using One‐Way ANOVA analysis. *p < 0.05, **p < 0.01, ***p < 0.001 compared with DED group. Non‐apt: non‐aptamer.

We subsequently investigated the efficacy of aptamer CsA liposomes (5 μg mL^−1^) in relieving dry eye conditions in comparison with 0.001% CsA solution. The CsA concentrations in 5 μg mL^−1^ liposomes and 0.001% CSA in the medium were 0.89 μg mL^−1^ and 10 μg mL^−1^, respectively. CsA of 0.001% was chosen based on its therapeutic effects on dry eye cell models as demonstrated previously.^[^ ^14^ ^]^ The cell viability after 24‐h treatment was first evaluated by an MTT assay. In Figure 5A, the hyperosmolarity medium significantly induced the death of HCECs compared to the control group. A prolonged 24‐h CsA (0.001%) treatment further suppressed cell viability in the hyperosmolarity medium (DED group). Such results implied the cytotoxicity of CsA, which was documented in the literature.^[^ ^36^ ^]^ The CsA liposomes inserted with aptamers did not cause cell death in HCECs compared to the DED group, suggesting encapsulation of CsA in liposomes reduced CsA cytotoxicity in HCECs.

The increase in inflammatory cytokines and chemokines in the tear fluid and the disruption of the tight junction of the corneal epithelium are commonly found in dry eye patients.^[^ ^37^ ^]^ Thus, we explored the prolonged effect of aptamer liposomes on anti‐inflammation and tight junction modulation of liposomes in the dry eye HCECs model. The cells were treated with liposomes or 0.001% CsA for 4 h in a hyperosmolarity medium, followed by 20‐h incubation in the treatment‐free medium. In Figure 5B–D, the hyperosmolarity medium caused a remarkable increase in the gene expression of inflammatory markers (IL‐6, IL‐8, and IL‐1β) in HECEs compared to the control group. Such increases could be reversed by 0.001% CsA treatment. Cornea‐S1 or Cornea‐S2 CsA liposomes, which acted like 0.001% CsA, significantly suppressed all inflammatory and tight junction markers in HCECs compared to the DED group. S2.2 CsA liposomes could only abolish hyperosmolarity medium‐induced IL‐8 and IL‐1β gene expressions, while non‐aptamer CsA liposomes showed no effect on these markers.

**FIGURE 6:**
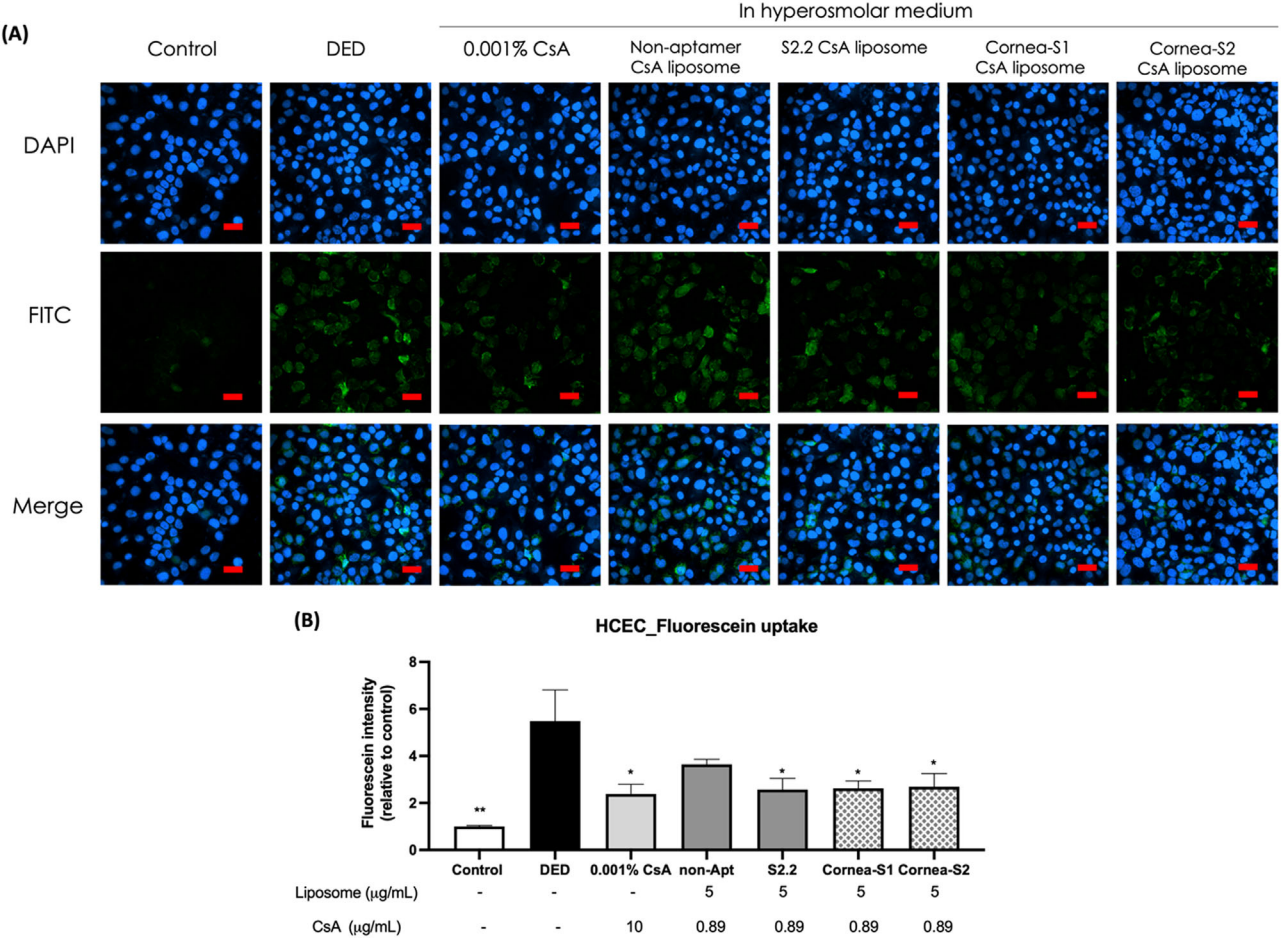
Cell permeability. (A) Fluorescence images of treated HCECs incubated with 1 mM fluorescein for 5 min. 4 h followed by 20‐h incubation in the culture medium after washing. The cell nucleus was stained with DAPI to give blue fluorescence and the green fluorescence (FITC) was from fluorescein. Scale bar: 60 μm. (B) Quantification of the fluorescent intensity. Data were shown as mean ± SEM and analyzed using One‐Way ANOVA analysis. ***p < 0.001 compared with the DED group.

Furthermore, the effect of these liposomes on tight junction modulation was confirmed using the cellular fluorescein uptake assay, a marker dye applied to the evaluation of tight‐junctional permeability of epithelial cells. In Figure 6A,B, a significant increase in fluorescein uptake was observed in HECEs under a hyperosmolarity medium, indicating tight junction disruption in epithelial cells under dry eye conditions. Like 0.001% CsA, S2.2, Corena‐S1 or Corena‐S2 CsA liposomes could significantly reduce the fluorescein uptake in HCECs under hyperosmolality medium, while the non‐aptamer CsA liposomes did not show a positive effect in this assay. Altogether, the liposomes inserted with aptamers performed better than those with non‐aptamer in managing dry eye conditions in HCECs. Also, the use of aptamer‐functionalized liposomes for delivery of CsA required a 10‐time lower concentration of CsA to achieve a similar effect as CsA alone, which might result from the aptamer‐assisted long retention time and uptake of liposomes.

### 2.5 Dry eye treatment in a rabbit model

To further assess the effectiveness of cornea‐targeting CsA liposomes in treating DED, an experimental rabbit model of dry eye induced by benzalkonium chloride (BAC) was employed. BAC is a commonly used chemical to induce dry eye symptoms, including tear film instability, epithelial cell apoptosis, and inflammation.^[^ ^38^ ^]^ Various concentrations of BAC were tested, ranging from 0.01% to 1.0%, as well as different frequencies of topical application (two to four times daily) and treatment durations (1−4 weeks), to evaluate their impact on the rabbit ocular surface. The evaluation methods included the Schirmer test, ocular surface staining, conjunctival impression cytology, and microscopic examination, as conducted in the study by Xiong et al.^[^ ^39^ ^]^

A 4‐week 0.1% BAC treatment could induce stable dry eye condition in rabbits and no severe corneal damage was observed.^[^ ^40^ ^]^ Thus, a 4‐week 0.1% BAC installation followed by a 3‐week topical treatment of Restasis® or liposomes was used in the present study. Corneal fluorescein staining, tear breakup time and tear production were measured before and after BAC installation and weekly throughout the 3‐week treatment period. The solutions intended for topical administration were found to have a pH value of 7.41, along with osmolarities of 289–300 mOsms L^−1^.

**FIGURE 7:**
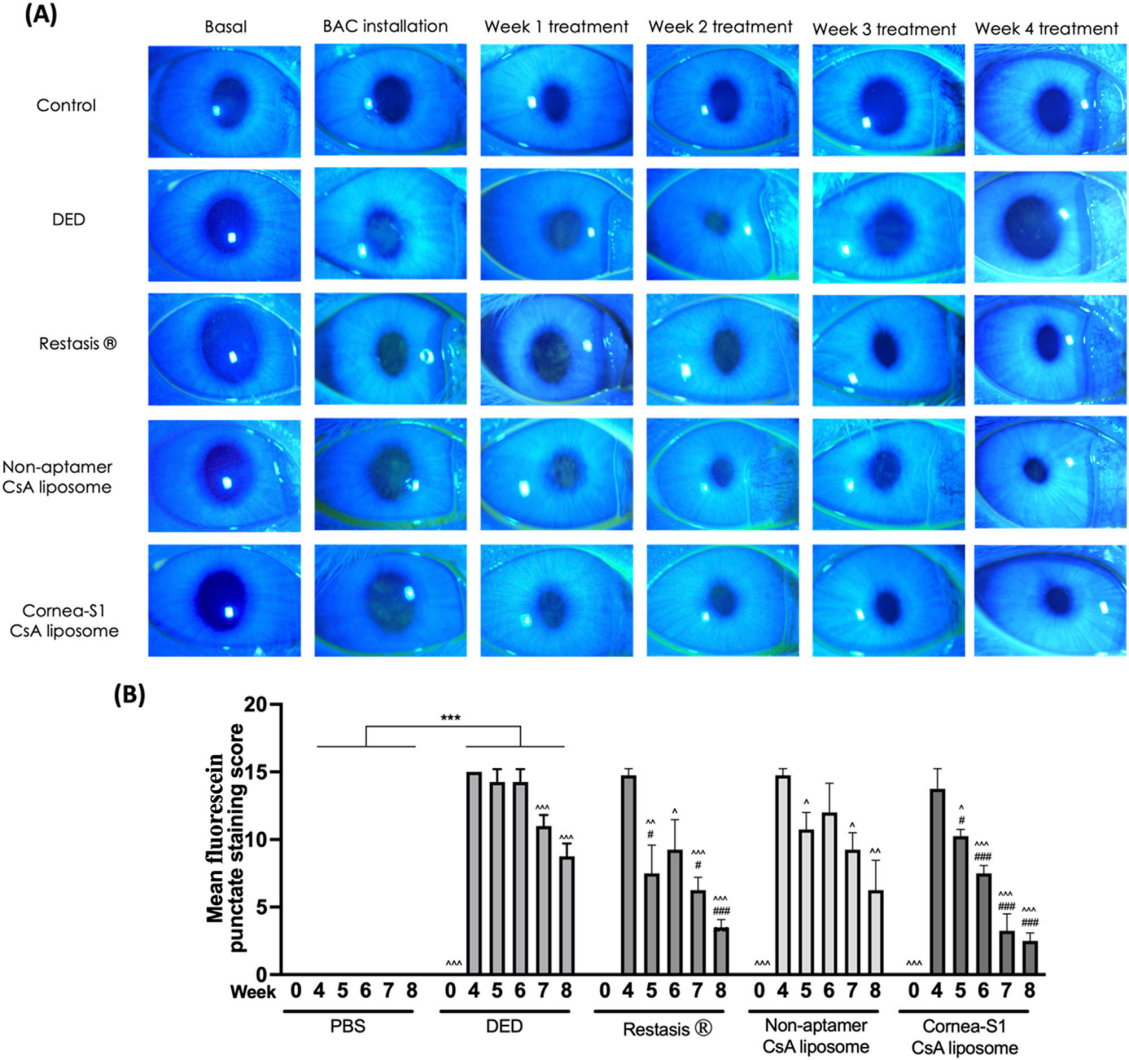
Fluorescence staining. (A) Representative images of fluorescein staining of the corneal epithelium of rabbits before and after BAC installation, as well as weekly following the initiation of treatments. (B) The mean fluorescein punctate staining scores were presented as mean ± SEM and analyzed using One‐Way ANOVA analysis. ***p < 0.001 compared between PBS and DED group; ^*p* < 0.05, ^^*p* < 0.01, ^^^*p* < 0.01 compared treatment to week 4, ^#^ *p* < 0.05, ^##^ *p* < 0.01 compared treatment to DED at a specific week.

Fluorescein staining on the cornea served as a clinical method to evaluate corneal integrity. Fluorescein is a water‐soluble dye that easily permeates and stains the corneal stroma in areas where the epithelium is missing or when the epithelial cells have lax intercellular junctions. This looseness in intercellular junctions is a symptom of DED, which can lead to cell death, damage to corneal barriers, and ultimately, corneal instability. This phenomenon has been observed in cases of DED.^[^ ^41^ ^]^ Figure 7A shows the appearance of the ocular surface under a slit‐lamp microscope. In Figure 7B, no fluorescein staining was observed at the baseline or in the control group throughout the experiment. In contrast, rabbits subjected to the 4‐week BAC installation exhibited a significant patch of fluorescein staining on the cornea, indicating the successful establishment of the dry eye model through increased dye permeability and damage to the epithelial layer. The administration of PBS to rabbits with DED did not lead to a significant reduction in fluorescein staining within the initial two weeks, only yielding a slight improvement in corneal integrity in regions 2 and 5 in week 3 (Figure S6a). This outcome strongly suggests the establishment of a stable DED model. In the treatment groups receiving Restasis® or Cornea‐S1, the previously pronounced staining was markedly diminished to residual staining or isolated dots following a 2‐week treatment period (Figure 7B). This observation implied that the impact of Cornea‐S1 CsA liposome on preserving corneal integrity was like that of Restasis®, thereby demonstrating comparable efficacy even at a lower CsA dose encapsulated in the liposomes. Furthermore, in rabbits receiving non‐aptamer‐bound CsA liposomes, residual fluorescein staining dots persisted on the cornea even after a 3‐week period. This observation underscored that the incorporation of corneal aptamers could enhance the effectiveness of CsA liposomes in DED management.

**FIGURE 8:**
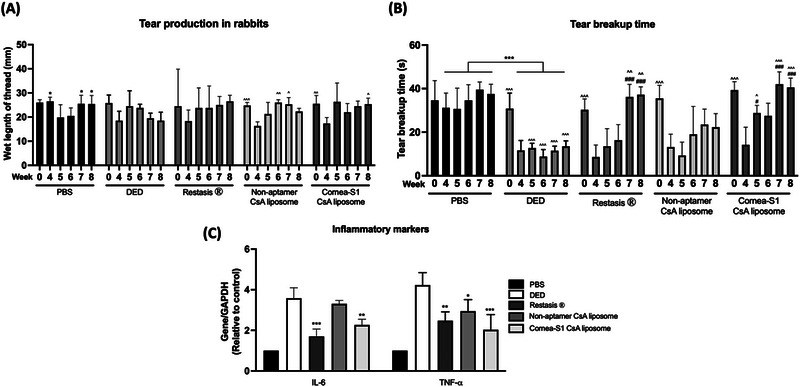
In vivo study results. (A) Tear break‐up time and (B) tear production in rabbits before and after BAC installation, as well as weekly following the initiation of treatments. Data are shown as mean ± SEM and analyzed using One‐Way ANOVA analysis. *p< 0.05, **p < 0.01, ***p < 0.001 compared between PBS and DED group; ^*p* < 0.05, ^^ *p* < 0.01, ^^^ *p* < 0.01 compared treatment to week 4, ^#^ *p*< 0.05, ^##^ *p* < 0.01 compared treatment to DED at specific week. (C) Gene expressions of IL‐6 and TNF‐α in cornea of treated rabbits. The data were normalized to GAPDH and relative to the control. Data are shown as mean ± SEM and analyzed using One‐Way ANOVA analysis. *p < 0.05, **p < 0.01, ***p < 0.001 compared with DED group, *n* = 4 eyes.

An indicator of evaporative DED is the disruption of tear film homeostasis. DED diagnosis often involves the employment of tear break‐up time (TBUT).^[^ ^42^ ^]^ Visualizing alterations in tear film distribution or corneal surface irregularities can be achieved through the implementation of the tearscope with a grid pattern, where the disruption of the grid indicates the break‐up of the tear film (Figure S5B,C).^[^ ^43^ ^]^ Figure 8 illustrates a notable reduction (approximately 60–70%) in TBUT among rabbits following 4 weeks of BAC installation, when compared to the baseline levels. This reduction indicates impaired tear film stability. However, this impairment was progressively and significantly alleviated within 3 weeks through the administration of Restasis® or Cornea‐S1 CsA liposomes, as evidenced by substantial restoration. Conversely, the DED group or rabbits treated with non‐aptamer CsA liposomes displayed limited or negligible restoration. Importantly, a statistically significant difference in TBUT was noted when comparing rabbits treated with Restasis® or non‐aptamer CsA liposomes to those subjected to Cornea‐S1 CsA liposomes. A decrease of approximately 25–35% in tear production was observed following the 4‐week BAC installation. Such changes were reversed by all three treatments. Although all three treatments led to restoration, statistical significance was not achieved. This lack of significance could potentially be attributed to significant individual variations, stimulated tear production during measurement, or the relatively small sample size. Besides, the gene expression of IL‐6 and TNF‐α was significantly increased in DED groups, which could be reversed by both Restasis^®^ and Cornea‐S1 CsA liposomes, while non‐aptamer CsA liposomes only reduced TNF‐α gene expression. These results indicated the anti‐inflammatory effect of CsA could be enhanced when it was loaded in Cornea‐S1 liposomes.

It is not surprising that Restasis^®^ showed favourable outcomes in the rabbit DED model.^[^ ^21^, ^44^ ^]^ The contribution of the present study was conducting tissue‐SELEX to identify new aptamers for the cornea that exhibited a comparable targeting effect to the mucin aptamer. The outcomes of our research presented convincing evidence that supported the viability of targeted corneal treatment by aptamer as an effective approach for addressing DED. The trends observed in our findings highlighted the need for further investigations, such as localized and systemic delivery of CsA, the distribution of aptamer‐functionalized liposomes on the ocular surface and the immune response of aptamer‐functionalized liposomes on the body.

## 3 CONCLUSIONS

To our best understanding, it is the first cornea‐SELEX, which can be considered a nice addition to aptamers derived from tissue‐SELEX work. Two cornea aptamers, Cornea‐S1 and Cornea‐S2, were selected and characterized. Cornea‐S1 and Cornea‐S2 could bind to pig cornea, and for HCECs they showed *K*_d_ values of around 361 and 174 nm, respectively. We further showed the insertion of Conena‐S1 or Conrea‐S2 aptamers with CsA‐loaded liposomes could increase the retention time, accelerate cellular uptake, and promote the therapeutic effect of CsA liposomes in HCECs and a rabbit DED model. Even though the efficacy of Cornea‐S1 and Cornea‐S2 liposomes in managing dry eye conditions was similar to that of the S2.2 mucin aptamer functionalized liposomes, Cornea‐S1 and Cornea‐S2 aptamers had lower *K*_d_ values compared to S2.2 aptamer. These cornea‐targeting aptamers will likely find application in targeted drug delivery to the cornea through the conjugation with various nanoparticles in the future. The delivered drugs do not have to treat eye‐related diseases, but the eyes can also be a delivery site for drugs to treat other types of diseases, such as brain disorders.^[^ ^45^ ^]^

## 4 MATERIALS AND METHODS

### 4.1 Tissue‐SELEX

Cornea‐sclera was isolated from pig eyeballs with dissecting scissors under a dissecting microscope. Corneal discs (5 mm) were cut out using a biopsy punch. The aptamer selection conditions in each round are illustrated in Figure S7. The DNA sequences are shown in Table S1. For each round of selection, the DNA library was first heated at 95°C for 5 min and cooled to room temperature. After washing the corneal disc with PBS twice, the disc was put in a well of a 96‐well plate and incubated with 0.5 mL DNA library in PBS with 1 mM Mg^2+^ on an orbital shaker at room temperature for 2 h. The cornea was washed nine times with PBS with 1 mM Mg^2+^ in new wells (2 mL/wash) on an orbital shaker to remove unbound DNA. The last three washes were collected as a background control. The bound DNA sequences were released by 3 mM ethylenediaminetetraacetic acid (EDTA) for 5 min. The supernatant was collected as the bound DNA. Both background control and EDTA‐released DNA were purified by a 3K MWCO centrifugal filter with Milli‐Q water 6 times and concentrated to 60 μL under centrifugation at 14,000 rpm 6 times (15 min/time). Real‐time polymerase chain reaction (RT‐PCR) was then performed to monitor the selection progress. Gel electrophoresis was also performed to confirm the optimal PCR cycles. The bound DNA sequences were amplified using a forward primer and a biotinylated reserve primer. Subsequently, the PCR products were concentrated and washed using a 10K MWCO centrifugal filter with sterile strand separation buffer, (1X PBS, pH 7.5) 10 times under centrifugation at 14,000 rpm for 10 times (10 min/time). To isolate bound DNA sequences, the PCR product was loaded onto a micro chromatography column packed with streptavidin agarose resin. The column was washed 10 times with 500 μL of strand separation buffer followed by the incubation of NaOH for 25 min to elute the bound DNA. An additional 0.2 M NaOH was added to the column followed by neutralization with 0.2 M HCl. The bound DNA sequences were concentrated and washed with Milli‐Q water and separation buffer using a 3K MWCO filter under centrifugation at 14,000 rpm four times (15 min/time). The DNA concentration was determined using a Spark microplate reader (Tecan) and subjected to the next round of selection. The bound DNA in round 12 was sequenced by the Illumina method (Table S2). Sequencing results were analyzed with Geneious Prime and Clustal Omega.

### 4.2 Animal studies

Ten male New Zealand White rabbits, weighing between 3 and 3.5 kg each, were procured from Charles River Laboratories. They were housed within the Centralized Animal Facility at the University of Waterloo, where they were subjected to a 12‐hour cycle of alternating light and darkness. The temperature was maintained at 23°C ± 2°C, with a relative humidity of 60% ± 10%. The entire experimental procedure was conducted by ethical guidelines and received approval from the Animal Care Committee under Application No. 44737. Upon a 2‐week acclimation period, eight rabbits were chosen at random to induce stable dry eye disease. This was achieved through the topical application of 0.1% benzalkonium chloride (BAC) at a dosage of 20 μL per installation. This protocol was repeated twice a day (at 10 a.m. and 5 p.m.) for the subsequent 4 weeks. The remaining two rabbits constituted the control group and received PBS through a similar administration schedule. Following this phase, all rabbits were randomly divided into five groups, each comprising two rabbits (4 eyes). Over the course of 3 consecutive weeks, the rabbits in each group received the designated treatments twice daily (at 9 a.m. and 5 p.m.) as follows: 1) Control Group: 20 μL of PBS; 2) DED Group (Model Group): 20 μL of PBS; 3) Positive Control Group: 20 μL of Restasis® 0.05% CsA eye drops; 4) Negative Control Group: 20 μL of sterile non‐aptamer CsA liposomes (0.5 mg mL^−1^) and 5) Experimental Group: 20 μL of sterile aptamer CsA liposomes (0.5 mg mL^−1^). In the Restasis® group, each application contained 10 μg of CsA, while in the liposomes group, the amount was reduced to 0.89 μg per application.

### 4.3 Fluorescein staining for corneal integrity

5 μL 1% fluorescein was dropped onto the ocular surfaces of the rabbits under sedation by an intramuscular injection of acepromazine (1 mg kg^−1^ body weight). The eyelids were blinked manually 2–3 times and then held open to distribute the fluorescein. After 5 min, the eye was flushed with 2 mL of sterile saline. The distributed fluorescein was observed with a slit lamp. Briefly, the cornea was divided into five areas (Figure S6A, central, superior, nasal, inferior, and temporal).^[^ ^46^ ^]^ The severity of corneal fluorescein staining was graded on a scale from 0 to 3. The scores from the five zones were then added up with a maximum score of 15.^[^ ^47^ ^]^

### 4.4 Tearscope analysis for tear break‐up time (TBUT)

Rabbits were sedated by the aforementioned procedure. TBUT was evaluated utilizing a handheld EasyTear® VIEW+ Tearscope with a grid pattern, in conjunction with the S4 OPTIK HR Elite Mega Digital Vision system. To simulate a blink, the eyelids were gently closed, initiating the TBUT assessment immediately upon the re‐opening of the eye. This measurement was repeated three times to ensure accuracy, and an average value was calculated for analysis.

### 4.5 Tear production

The aqueous tear production was measured with Schirmer Tear Strips (SPORTS WORLD VISION). The strips were placed inside the margin of the nictitating membrane of the lower eyelid of the rabbit for 5 min and the wetting length of the strip was measured as an indication of tear production.

### 4.6 Gene expression of inflammatory markers

The cornea of rabbits were collected and homogenized. RNA was extracted from the cells using Isol‐RNA Lysis Reagent, and reverse transcription was performed using the iScript™ cDNA Synthesis Kit according to the manufacturer's instructions. The expression levels of target genes, including interleukin‐6 (IL‐6), tumor necrosis factor alpha (TNF‐α) and glyceraldehyde‐3‐phosphate dehydrogenase (GAPDH), were measured using SsoFast EvaGreen with specific primer pairs (Table S1). The gene expression was normalized to GAPDH. Additional methods are supplied in Supporting Information.

## AUTHOR CONTRIBUTIONS

Ka‐Ying Wong contributed to conducting the experiments, analyzing the data, and preparing the manuscript. Yibo Liu contributed to designing the experiments and providing advice. Man‐Sau Wong contributed to the conceptualization of the topics and provided advice. Juewen Liu contributed to the conceptualization of the topic, providing advice, and revising the manuscript. All authors have approved the final version of the manuscript.

## CONFLICT OF INTEREST STATEMENT

The authors declare no conflict of interest.

## Supporting information

Supporting Information

## Data Availability

The data that support the findings of this study are available from the corresponding author upon reasonable request.
