# Supplementary material for: Cornea‐SELEX for aptamers targeting the surface of eyes and liposomal drug delivery
Source: Exploration (Beijing). 2024 Feb 9;4(4):20230008. doi: 10.1002/EXP.20230008 (PMC11335462; doi:10.1002/EXP.20230008)
Supplement: Supplementary file 1 — Supporting Information [file EXP2-4-20230008-s001.pdf]

Supporting information

Cornea-SELEX for Aptamers Targeting the Surface of Eyes and Liposomal Drug Delivery

Ka-Ying Wong[1,2], *Yibo Liu*[1,2], *Man-Sau Wong*[2,3,4], *Juewen Liu**[1,2]

[1]Department of Chemistry, Waterloo Institute for Nanotechnology, University of Waterloo, Waterloo, ON, N2L 3G1, Canada;

[2]Centre for Eye and Vision Research (CEVR), 17W Hong Kong Science Park, Hong Kong;

[3]Department of Food Science and Nutrition, The Hong Kong Polytechnic University, Hung Hom, Kowloon, Hong Kong

[4]Research Center for Chinese Medicine Innovation, The Hong Kong Polytechnic University, Hung Hom, Kowloon, Hong Kong SAR, PR China

Table of Contents

Scheme S1	12-cycle tissue-SELEX for pig cornea ……………………………………	2
Figure S1	The 8 most abundance sequences in the sequencing results……………..	3
Figure S2	S2.2 aptamer binding to HCECs……..…...……...…...…........................	4
Figure S3	Aptamer binding to HCECs upon disruption of surface protein………....	5
Figure S4	Quantification of free aptamer and aptamer on liposome ………………	6
Figure S5	Time-dependent cellular uptake of liposome……..………………..….....	7
Figure S6	Evaluation of fluorescein punctate staining and tear film..………....….	8
Figure S7	SELEX conditions and progression………………...…....…………….	9
Table S1	Oligo sequences …………………………....…....….………………..	10
Table S2	Sample preparation for DNA sequencing..…......…...…....………..….	11
Additional experimental methods……………………………………………………….		12
References……………………………………………………………………………….		14

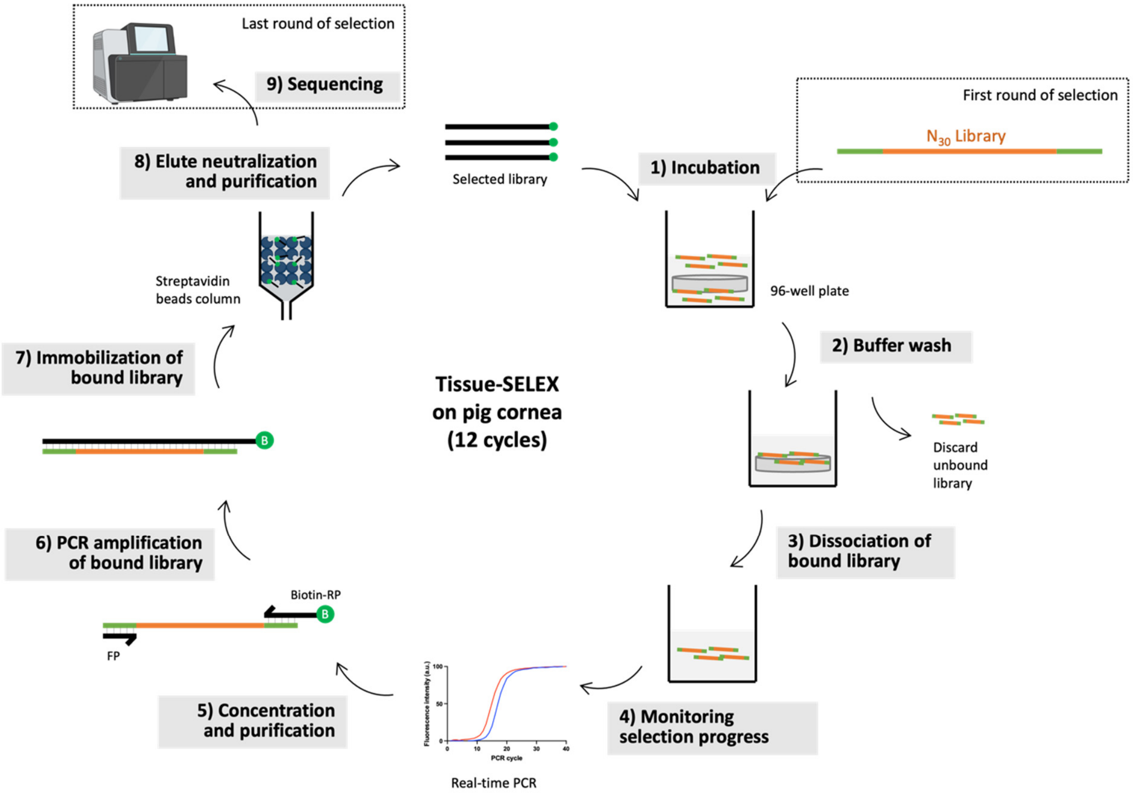

Scheme S1. The tissue-SELEX method using pig cornea as a target. A corneal disc was incubated in the DNA library containing an N30 random region flanked by two constant regions for primer binding. The bound DNA was released by adding EDTA, collected and amplified by PCR for the next round of selection.

	Sequence (5'-3')	Counts
Family 1		50.8%
Cornea-S1	GACGACGGCAAGGGGAAAGTGGTCGTAATCACGACGGTCGTC	15,937
Family 2		6.19%
Cornea-S2	GACGACTTATGCTTGGGACCTGATCCGGACTACGGTGTCGTC	1,941
Family 3		4.28%
Cornea-S3	GACGACGACAAACCAGCGGCTTGTGAGAATGCGTTCGTCGTC	588
Cornea-S6	GACGACGACAAACCAGCGGCTTGTGAGAATGCGTTCGCCGTC	128
Cornea-S7	GACGACGACAAACCAGCGGCTTGTGAGAATGCGGTCGTCGTC	60
Family 4		0.91%
Cornea-S4	GACGACAAGTTCCCCGGCAGGGCCATTGTGAGAAAACGTCGTC	285
Family 5		0.55%
Cornea-S5	GACGACAGCGAGCCGGAGGGAATGAGAACGAGTGGAGTCGTC	174
Family 6		0.11%
Cornea-S8	GACGACTCTACTTTACATGGTCAATGGGCGTGCGGTGTCGTC	36

Figure S1. The 8 most abundant sequences in the sequencing results. The sequences have more than 0.1% counts in the sequencing results were analysed and grouped in 6 families after sequence alignment.

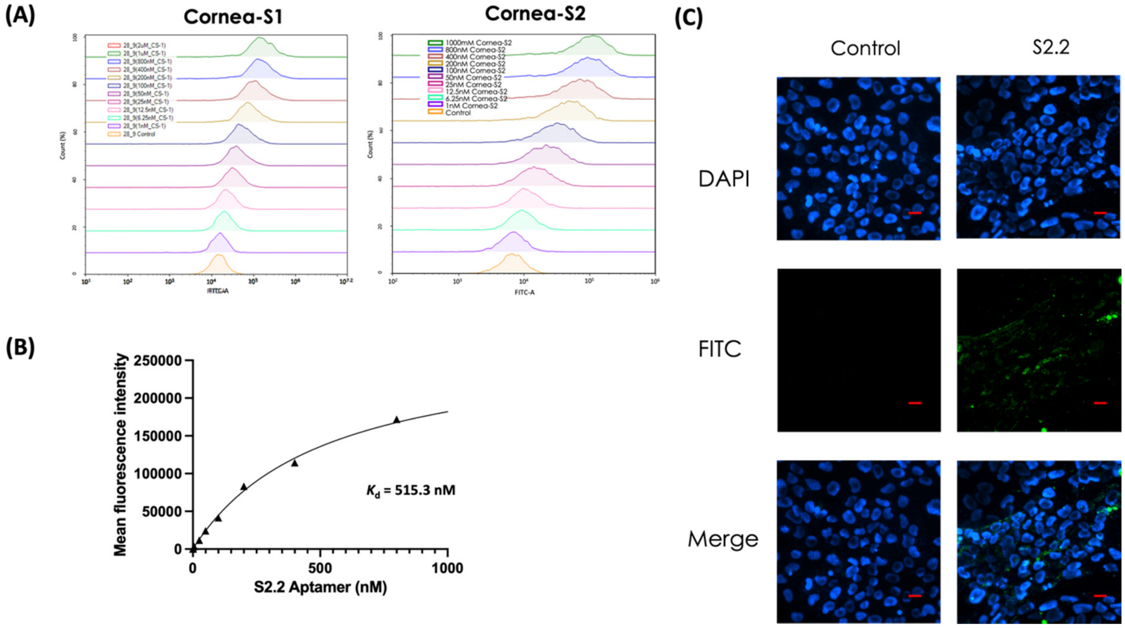

Figure S2. Assessment of binding properties of FAM-labelled S2.2 aptamers to HCECs by flow cytometry. (A) Flow cytometry data for FAM-labelled Cornea-S1 and Cornea-S2 aptamer in HCECs. (B) The K_d value of S2.2 aptamers to HCECs was calculated based on the FAM fluorescence intensity determined by flow cytometry. (C) Binding of S2.2 aptamer in HCECs. The binding ability of FAM-labelled Cornea aptamers in HCECs. The cell nucleus was stained with DAPI to give blue fluorescence and the green fluorescence was from FAM-labelled aptamers. Scale bar: 20 μm.

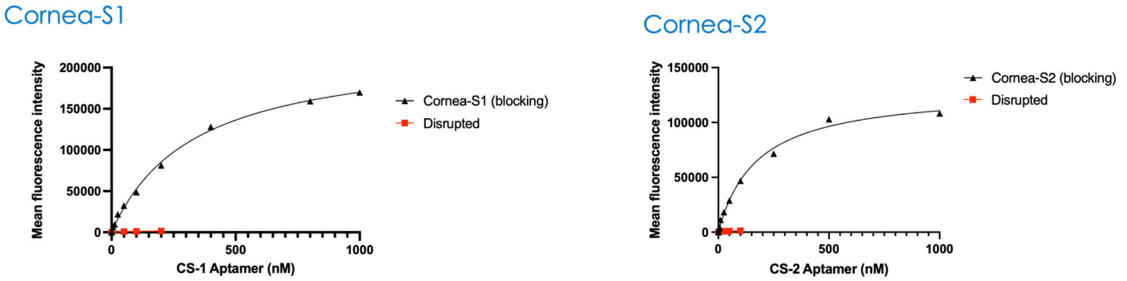

Figure S3. Assessment of binding properties of FAM-labelled aptamer to surface protein-disrupted HCECs by flow cytometry. HCECs were detached from the culture flask by 0.25% trypsin and 0.1 mg mL^{-1} proteinase K followed by incubation with FAM-labelled cornea aptamers. The mean fluorescence intensities of (A) Conrea-S1 and (B) Cornea-S2 in intact cells (black curve) and disrupted cells (red curve) were measured by flow cytometry.

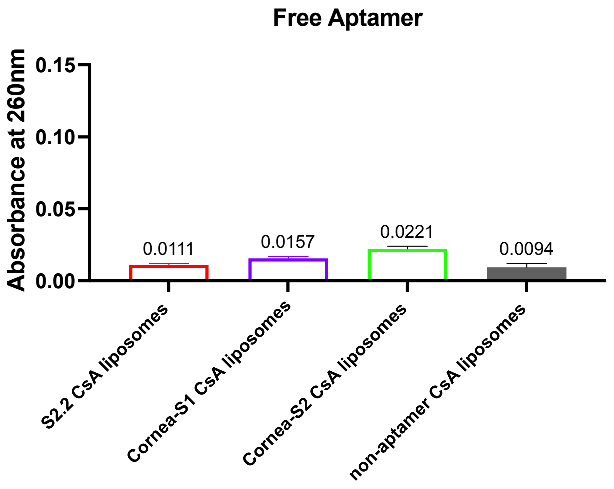

Figure S4. Absorbance of free aptamer at 260 nm after aptamer inserted to the liposome.

The absorbance measurements at 260 nm were taken for the free aptamer in four different samples: CsA liposomes inserted with S2.2, Cornea-S1, Cornea-S2, and non-aptamer. The values obtained were 0.0111, 0.0157, 0.0221, and 0.0094, respectively. By using the Beer-Lambert law with a path length of 0.05 cm (measured using a NanoQuant Plate™ in Tecan Spark) and the extinction coefficients of S2.2, Cornea-S1, Cornea-S2, and non-aptamers (200201, 459701, 433301, and 272201, respectively), the amount of free aptamers present in a 200 µL reaction were calculated. The results showed that the S2.2 CsA liposomes, Cornea-S1 CsA liposomes, Cornea-S2 liposomes, and non-aptamer CsA liposomes contained 221.7 pmol, 96.5 pmol, 204.1 pmol, and 138 pmol of free aptamers, respectively. After subtracting the amount of free aptamers from the initial amount of aptamers (1000 pmol), the approximate amount of inserted aptamers present were estimated. The results indicated that the S2.2 CsA liposomes, Cornea-S1 CsA liposomes, Cornea-S2 liposomes, and non-aptamer CsA liposomes contained 779 pmol (78% of the initial amount of aptamer), 904 pmol (90%), 796 pmol (79.6%), and 862 pmol (86.2%) of inserted aptamers, respectively.

To assess the DNA density on the liposomes, we performed calculations to determine that 20 µL of 5.0 mg mL^{-1} liposome could adsorb approximately 835 pmol DNA on average. By analyzing the molar concentration of phospholipid in the liposomes (6.34 mM) and the total mole of phospholipids (6.34 µmol), we estimated that each liposome contained approximately 105,000 phospholipid molecules, assuming the size of the phospholipid head group was 0.6 nm. However, we did not take into account the effect of cholesterol on the average size of the phospholipid head groups in this estimation. Moreover, based on the DLS results, we found that the average particle size of the 20% CsA loading group was 106 nm. Using these values, we calculated that the amount of liposomes was approximately 1.21 pmol (6.34 mM × 20 µL / 105,000 = 1.21 pmol). Thus, we estimated that each 106 nm liposome contained approximately 690 DNA molecules on average.

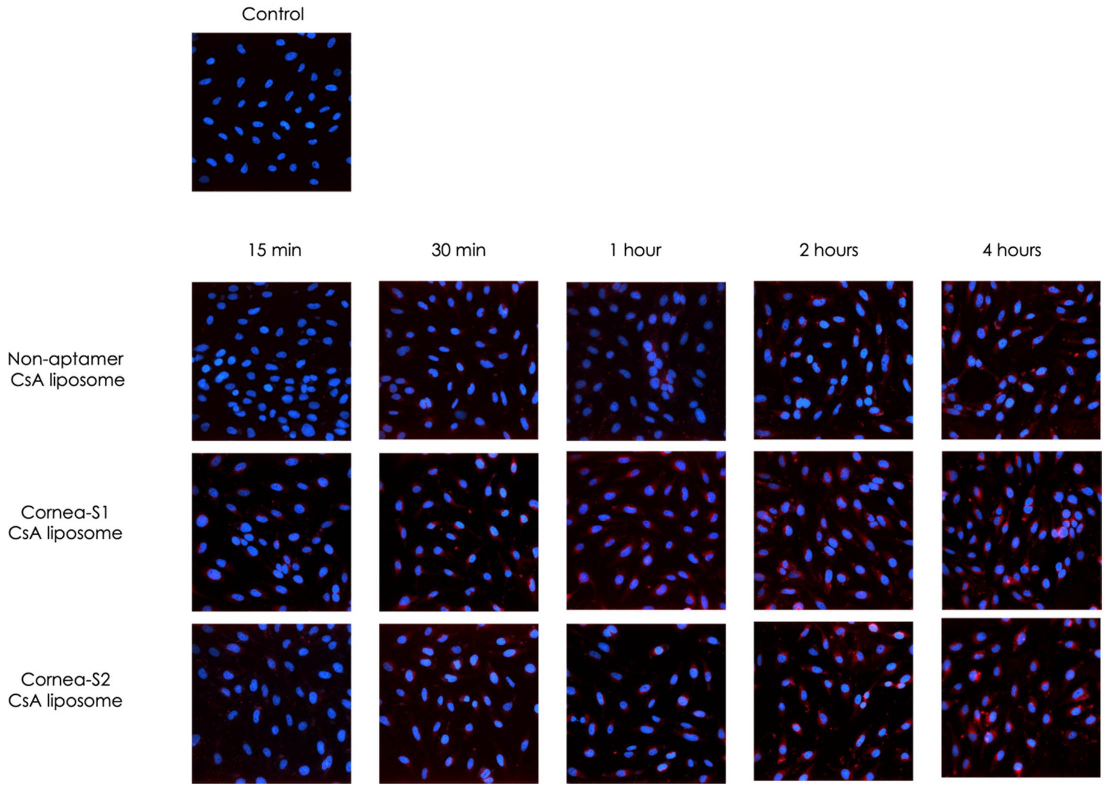

Figure S5. Time-dependent cellular uptake of liposomes. HECEs were treated with non-aptamer, Corena-S1 or Corean-S2 CsA liposomes for 15 min, 30 min, 1 h, 2 h, or 4 h in a hyperosmolarity medium. The cellular uptake of liposomes was studied using fluorescence microscopy. The cell nucleus was stained with DAPI to give blue fluorescence and the red fluorescence was from Rhod PE. Scale bar: 20 μm. Non-apt: non-aptamer. The results indicated the presence of Conrea-S1 and Cornea-S2 aptamers allowed cellular uptake of liposome in 15 min and accumulation of liposome in 4 h in HCECs, while the non-aptamer group required longer incubation time (30 min) to have observable liposome uptake.

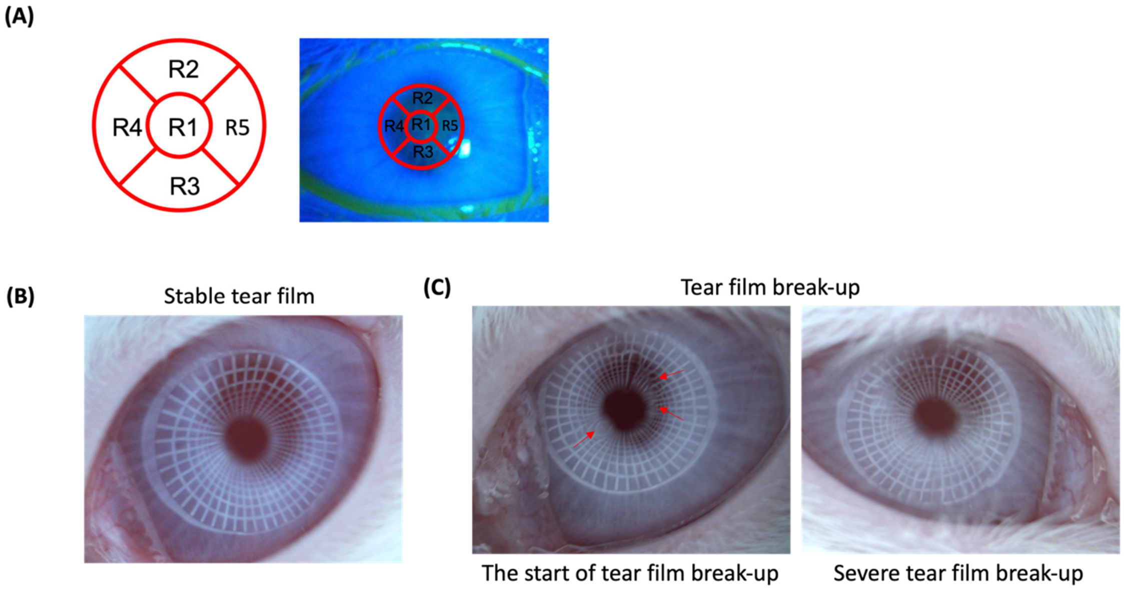

Figure S6. Evaluation of fluorescein punctate staining and tear film. (A) The cornea was divided into five regions for fluorescein punctate assessment. The images of (B) a stable tear film and (C) unstable tear films with tear film break-up (arrow) were observed under tearscope with a grid pattern.

(A)

No. of round	N_{30} Library (pmol)
1	500
2	400
3	300
4	200
5	100
6	100
7	100
8	100
9	100
10	100
11	100
12	100

(B)

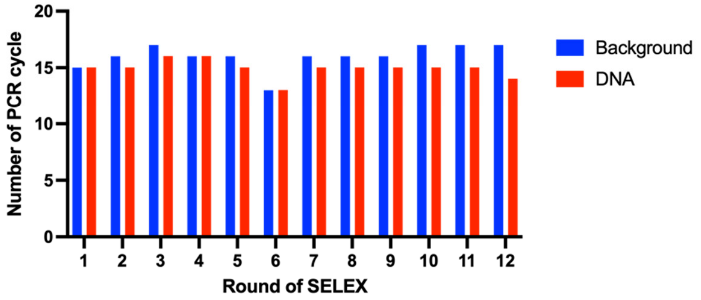

Figure S7. SELEX condition and progress. (A) List of library concentrations in each round. (B) SELEX progress monitoring by RT-PCR results. The y-axes is the number of RT-PCR cycles it took to reach 50% of the maximum fluorescence signal or the midpoint cycle.

Table S1. Oligo sequences used in this work.

Oligo	Sequence (5′->3′)
**IL-1β forward primer**	CCTGTCCTGCGTGTTGAAAGA
**IL-1β reverse primer**	GGGAACTGGGCAGACTCAAA
**IL-6 forward primer**	CACAGACAGCCACTCACCTC
**IL-6 reverse primer**	TTTTCTGCCAGTGCCTCTTT
**IL-8 forward primer**	TTTCAGAGACAGCAGAGCACACAA
**IL-8 reverse primer**	CACACAGAGCTGCAGAAATCAGG
**GAPDH forward primer**	GAAGGTGAAGGTCGGAGTC
**GAPDH reverse primer**	GAAGATGGTGATGGGATTTC
**Library**	GGAGGCTCTCGGGACGACN30GTCGTCCCGACT CTATGATGACTGT
**Column-binding strand**	GTCGTCCCGAGAGCCATA-/3Biosg/
**SELEX forward primer**	GGAGGCTCTCGGGACGAC
**SELEX reverse primer**	ACAGTCATCATAGAGTCGGGACG
**SELEX Biotin-reverse primer**	/5Biosg/ACAGTCATCATAGAGTCGGGACG
**Cornea-S1-cho**	GACGACGGCAAGGGGAAAGTGGTCGTAATCAC GACGGTCGTCTTTTT-Chol
**Cornea-S2-cho**	GACGACTTATGCTTGGGACCTGATCCGGACTAC GGTGTCGTCTTTTT-Chol
**S2.2-cho**	GCAGTTGATCCTTTGGATACCCTGGTTTTT-Chol
**B2-cho**	ACTTCAACATCTAGCTGGTGG-Chol
**FAM-Cornea-S1**	GACGACGGCAAGGGGAAAGTGGTCGTAATCAC GACGGTCGTC/36-FAM/
**FAM-Cornea-S2**	GACGACTTATGCTTGGGACCTGATCCGGACTAC GGTGTCGTC/36-FAM/
**Biotin-Cornea-S1**	/5Biosg/GACGACGGCAAGGGGAAAGTGGTCGTA ATCACGACGGTCGTC
**Biotin-Cornea-S2**	/5Biosg/GACGACTTATGCTTGGGACCTGATCCGG ACTACGGTGTCGTC
IL-6 Forward	GTCTTCCTCTCTCACGCACC
IL-6 Reverse	TGGGCTAGAGGCTTGTCACT
TNF-α	GTCTTCCTCTCTCACGCACC
TNF-α	TGGGCTAGAGGCTTGTCACT

Table S2. Sample preparation for DNA sequencing

Oligo	Sequence (5′->3′)
**P5-503**	AATGATACGGCGACCACCGAGATCTACACTAT CCTCTACACTCTTTCCCTACACGACGCTCTTCC GATCTACAGTCATCATAGAGTCGGGACG
**P7-704**	CAAGCAGAAGACGGCATACGAGATGCTCAGGA GTGACTGGAGTTCAGACGTGTGCTCT TCCGA TCTGGAGGCTCTCGGGACGAC

The enriched pool from round 12 was used as the DNA template and was subjected to another PCR reaction using forward primer (P5-503) and reverse primer (P7-704) containing unique index sequences were used. Agarose gel was used to purify the PCR products subjected to gel extraction using a small DNA fragment extraction kit (IBI Scientific). The concentration of the purified DNA was quantified. The samples were submitted to McMaster University Genomics Facility for Illumina sequencing.

Additional experimental methods

Chemicals

Porcine eyeballs were obtained from a local market (Highland Packers, Hamilton). Phospholipids were purchased from Avanti Polar Lipids. All aptamers and primers were purchased from Integrated DNA Technologies, and their sequences are listed in **Table S1**. Cyclosporine, Fluoromount™ Aqueous Mounting Medium, proteinase K, and deoxyribonucleic acid from salmon sperm were purchased from Sigma-Aldrich. 3K (3K MWCO and 10K MWCO Amicon Ultra-0.5 mL Centrifugal Filters were purchased from Millipore Sigma. Bovine serum albumin was purchased from HyClone. Streptavidin agarose resin and cell culture-related chemicals including medium, serum, and antibiotics were purchased from Fisher Scientific Inc. Culture flasks and Hoechst 33342 solution were purchased from Thermo Fisher. Sodium chloride, Isol-RNA Lysis Reagent, and MTT were purchased from VWR. PBS, iScript™ cDNA Synthesis Kit, Micro bio-spin chromatography columns, and SsoFast EvaGreen supermixes were purchased from Bio-Rad. Glass bottom dishes were purchased from Greiner Bio-One. Milli-Q water was used to prepare all buffers, solutions, and suspensions. All buffers and solutions were prepared with Milli-Q water.

Synthesis of CsA liposomes and CsA loading efficacy

The synthesis of liposomes and the assessment of CsA loading were performed based on our previous methodology.[1]

Aptamer functionalization and analysis

The top two abundance aptamers in the sequencing results were studied, namely Cornea-S1 aptamer (5′-GACGACGGCAAGGGGAAAGTGGTCGTAATCACGACGGTCGTC-Chol) and Cornea-S2 (5′-GACGACTTATGCTTGGGACCTGATCCGGACTACGGTGTCGTC-Chol). To insert the aptamers with unloaded liposome or CsA liposome, 10 μL of aptamer was mixed with liposome suspension (5 mg mL^{-1}), PBS, and NaCl (pH 7.5, 5 M) at 1:2:2:2 ratio in Milli-Q water to achieve a final volume of 200 μL. After 24-h incubation, the free aptamer was separated from the liposome by ultracentrifugation (Beckman Optima TLX Ultracentrifuge) at 120,000 rpm for 30 min. The concentrations of free aptamer were measured by spectrophotometer (Tecan Spark). The cornea-S1 liposomes or cornea-S2 liposomes were resuspended with 20 μL of PBS (Liposome concentration at 5 mg mL^{-1}). A S2.2 aptamer targeting mucin 1 (S2.2: 5′-GCAGTTGATCCTTTGGATACCCTGGTTTTT-Chol) or a non-aptamer (B2: 5′-ACTTCAACATCTAGCTGGTGG-Chol) was inserted with the CsA liposome as the positive or negative control, respectively.

Dry eye cell model

HPV-immortalized HCECs were gifted from Dr. Maud Gorbet at the University of Waterloo. The culture method was based on Dr. Gorbet's method with a few modifications.[2] The cells (passages 6-10) were maintained in DMEM/F12 medium supplemented with 1% FBS and 1% penicillin/streptomycin at 37°C, 95% humidity, and 5% CO2. The cell medium was changed every 2 days. To establish an *in vitro* DED model, sodium chloride was added to the culture medium to increase the osmolarity of the medium by 200 mOsM.

Cell viability

HCECs were seeded in 96-well plates at 12,000 cells/well and incubated for 24 h. Afterward, HCECs were treated with vehicle, CsA (0.001% in medium), aptamer or non-aptamer CsA liposomes under serum-free hyperosmolarity medium for 24 h. HCECs cultured in the isosmotic medium with a vehicle were used as a control. Cell viability was measured using MTT assay, following the manufacturer's instructions.

Flow cytometry

To investigate the binding affinity of FAM-labelled aptamer or aptamer liposomes to the cells, HCECs were harvested using a scrapper or detached from the flask by dissociation solution (0.25% trypsin and 0.1 mg mL^{-1} proteinase K). The cells were then incubated with blocking buffer (1% BSA, 0.1mg mL^{-1} Salmon sperm DNA in PBS) for 30 min at room temperature. After washing with PBS under centrifugation, 3×10^5 cells were incubated with pre-heated Cornea-S1 or Cornea-S2 aptamers or aptamer liposomes at various concentrations in PBS for 30 min at room temperature. After incubation, the cells were rinsed three times with 500 µL of PBS and then resuspended in 200 µL of PBS. The fluorescence signal was analyzed with a NovoCyte Flow Cytometer (Agilent Technologies). The equilibrium dissociation constants (K_d) value of each aptamer or aptamer liposome was obtained by GraphPad Prism 9 according to $Y = Bmax*X (K_d + X)^{-1}$.

Aptamer binding and uptake of liposome in cells

HCECs were seeded on glass bottom culture dishes at 10,000 cells/compartment for 24 h. To test the binding of the aptamer to cells, HCECs were incubated with FAM-labelled T30 DNA sequence (negative control), Cornea-S1, or Cornea-S2 aptamers for 30 min. As for liposome uptake in cells, HCECs were incubated with CsA liposomes inserted with non-aptamer, S2.2 aptamer, Cornea-S1 aptamer, or Cornea-S2 aptamer (5 µg mL^{-1} liposomes) for 4 h with or without 20-h post-incubation in serum-free hyperosmolarity medium. HCECs cultured in the isosmotic medium were used as a control. After incubation, the cells were washed 2 times with ice-cold PBS and were then stained with 1 µg mL^{-1} Hoechst 33342 in PBS for nucleus staining for 1 min at RT. Fluorescence images were captured at the mid-plane of cells (magnification: 400×) by Nikon Eclipse Ti Inverted Research Microscope. The excitation wavelengths of Hoechst, Rh-PE, and FAM were 361 nm, 560 nm, and 498 nm respectively.

Real-time PCR

HCECs were seeded in 6-well plates at 250,000 cells/well and incubated for 24 h. Afterward, HCECs were treated with CsA (0.001% in medium), aptamer, or non-aptamer CsA liposomes (5 µg mL^{-1} liposomes) under serum-free hyperosmolarity medium for 4 h. After incubation, the cells were further incubated in a serum-free hyperosmolarity medium for 20 h. The reverse transcription and real-time PCR were performed as described previously. The sequences of specific primer pairs were listed in Table S1.

Cell membrane permeability

HCECs were seeded on glass bottom culture dishes at 10,000 cells/compartment for 24 h. The cells were treated the same as in real-time PCR. After treatment, the cells were rinsed 2 times with PBS. The membrane integrity was studied using a fluorescein uptake assay. The cells were incubated with fluorescein (1 mM) in the medium for 5 min followed by the 1 µg mL^{-1} Hoechst 33342 in PBS for 1 min at RT. The fluorescein uptake was captured as mentioned. The excitation wavelength of Hoechst and fluorescein were 361 nm and 498 nm, respectively. The overall intensities of the fluorescent signals were also quantified using the corresponding Nikon system.

Statistical analysis

For MTT, RT-PCR, flow cytometry, and cell cellular uptake assays, the data were reported as mean ± SEM. Inter-group differences were analyzed by one-way ANOVA with Tukey's as a post hoc test. A value of $P< 0.05$ was considered statically significant. All graphs in this study were plotted by using GraphPad Prism Version 9.0.

References

[1] K.-Y. Wong, Y. Liu, L. Zhou, M.-S. Wong, J. Liu, *J. Mater. Chem. B* **2023**, *11*, 4684.
[2] S. Molladavoodi, M. Robichaud, D. Wulff, M. Gorbet, *PLOS ONE* **2017**, *12*, e0178981.
